# The oriental latrine fly *Chrysomya megacephala* (Fabricius, 1794) (Diptera: Calliphoridae) as a new forensic indicator in SW Europe

**DOI:** 10.1007/s00414-025-03489-z

**Published:** 2025-04-12

**Authors:** Anabel Martínez-Sánchez, Tania Ivorra, Leticia C. Roberts, Salvador Giner, Luisa M. Beringola, Pedro M. Cano, Santos Rojo

**Affiliations:** 1 Department of Environmental Sciences and Natural Resources, University of Alicante, PO Box 99, 03080 Alicante, Spain; 2 Department of Parasitology, Faculty of Medicine, University Malaya, 50603 Kuala Lumpur, Malaysia; 3 Institute of Legal Medicine of Alicante, Pathology Service, Generalitat Valenciana, 03006 Alicante, Spain; 4 National Institute of Toxicology and Forensic Sciences, Ministerio de la Presidencia, Justicia y Relaciones con las Cortes, 28232 Las Rozas de Madrid, Madrid, Spain

**Keywords:** Blow fly, Exotic species, Forensic entomology, Human corpses, Postmortem interval, Spain

## Abstract

The oriental latrine fly, *Chrysomya megacephala* (Fabricius, 1794), is a potential forensic indicator in Mediterranean countries due to its first report in southwestern Europe in 1998. It was first recorded in Alicante (southeastern Spain) and is expanding across the Iberian Peninsula. Until now, this synanthropic species of forensic importance had been captured in baited traps and on pig carcasses in Europe, but never on human corpses. After reviewing all cases studied at the Institute of Legal Medicine of Alicante and the National Institute of Toxicology and Forensic Sciences in Madrid, *C. megacephala* was recorded for the first time in seven Spanish cases. The identification of material collected during autopsies revealed its scarcity compared to other species in the sarcosaprophagous Diptera community. In all cases, *C. megacephala* larvae coexisted with thermophilous necrophagous blow fly *Lucilia sericata*. Other coexisting species included the calliphorids *Calliphora vicina* and *Chrysomya albiceps,* and the muscid *Synthesiomyia nudiseta*. *Chrysomya megacephala* was collected in various coastal localities in Spain, particularly along the southern and Mediterranean coasts. Most cases shared common characteristics, including the time of year (summer), domestic indoor habitats, and the decomposition stage (active decay). The relevance of these findings is discussed in relation to the importance of taxonomy in identifying this exotic species and its role as forensic entomological evidence in Europe. Given the impact of climate change, the species composition and biology of the sarcosaprophagous Diptera community, particularly in the Mediterranean basin, must be continuously studied and reviewed.

**Supplementary Information:**

The online version contains supplementary material available at 10.1007/s00414-025-03489-z.

## Introduction

Three medically and veterinary important species of *Chrysomya* Robineau-Desvoidy, 1830 (Diptera, Calliphoridae) are established in Europe: *C. albiceps* (Wiedemann, 1819), the most common and a native species; *C. putoria* (Wiedemann, 1830), exclusively present in the Canary Islands; and *C. megacephala* (Fabricius, 1794), recorded exclusively in the Iberian Peninsula and Malta, and coexisting with the other two in the Canary Islands [1–3]. The distributions of these species have been expanding over time. *Chrysomya albiceps*, originally from Africa, has been found in Asia, Europe, and South and North America, while the African *C. putoria* was introduced to South America in 1975 [4–7]. *Chrysomya megacephala*, originally distributed across Oriental and Australasian regions, has significantly expanded its range and is now established in all regions of the world, including the Ethiopian, Palearctic, Australian, Neotropical, and Nearctic regions, due to globalisation [8]. The eusynanthropic lineage of *C. megacephala*, originating from New Guinea, is likely the most widespread. This lineage is restricted to densely populated urban and suburban areas because of its attraction to a wide variety of human foods, human and livestock faeces, and carrion [9].

The oriental latrine fly, *C. megacephala*, is the most recently discovered necrophagous blow fly species in inland Europe. Over the last 25 years, it has been captured in the Iberian Peninsula, although it was already known in the Canary Islands (Spain) [10, 11]. It was first recorded in Alicante province (southeast Spain) in 1998 [2]. Subsequently, it was captured in Portugal and Malta, and its presence and expansion northward were confirmed with records in the Basque Country (northern Spain) [3, 12, 13]. New records currently place this species in Barcelona, Valencia, Granada, Cádiz, and Sevilla in the northeast, east, and south of Spain, respectively [14]. To date, adults have been collected on flowers and on bait such as fish, liver, or chicken, while larvae have been found on animal carcasses in urban, natural, and peri-urban areas in Europe, but never on human corpses.

To identify the two *Chrysomya* species present in continental Europe, morphological keys and larval descriptions are available in published studies from Europe and Africa [15–17]. Mature larvae of *C. megacephala* can be differentiated from other blow flies present in Europe, by their smooth bodies, segment III in the thorax with dorsolateral spines often featuring serrated tips (bicuspids and tricuspids), at least partly sclerotised oral sclerites, and posterior spiracles positioned close together with an incomplete peritreme. Adults can be identified using the identification keys established by Lutz et al. [18] and González-Mora & Peris [19], which include the three European *Chrysomya* species (*C. megacephala*, *C. putoria*, and *C. albiceps*). The oriental latrine fly is a green–blue metallic blow fly with haired stem veins above, transparent anterior wing margins, eyes with enlarged upper facets in males [8], orange genae, and brown anterior thoracic spiracles.

Synanthropic and necrophagous, with a widely pantropical distribution, *C. megacephala* is of interest in medical and veterinary entomology, as well as being a coloniser of vertebrate carrion and a forensic indicator in warm regions of the world. It is also known as a facultative agent of myiasis in humans and livestock [8, 20–22]. In addition, *C. megacephala* is widely recognised for its implications in public health due to its potential as a mechanical vector of human pathogenic bacteria [23] and as a pest in the salted-fish industry [24].

In forensic entomology, *C. megacephala* is an important indicator species as an early coloniser of vertebrate carrion. The application of knowledge about its development from egg to adulthood to specimens collected from remains is used to estimate the minimum postmortem interval (minPMI) when assuming colonisation occurred after death. It has been reported on human corpses from Asia [25], South America [26], Africa [27], and North America [28]. Differences in life cycle span have been observed depending on temperature, type of substrate, and population origin, and these factors must be considered when estimating the minPMI [29–31]. Currently, maggot age estimation depends on developmental data, sometimes derived from non-local populations. However, it is vital to emphasise the importance of advancing knowledge of the biology of local populations to obtain the most accurate estimations [32, 33]. For *C. megacephala*, data on annual activity, growth curves, isomorphen diagrams, and accumulated degree-days from original specimens have been recently published [34].

To evaluate the role of *C. megacephala* in Europe, given its potential for increase as forensic evidence, the aims of the present study were (i) to identify specimens of this blow fly collected during autopsies in different Spanish locations, provided by the Institute of Legal Medicine of Alicante (ILMA) and the National Institute of Toxicology and Forensic Sciences (INTCF); (ii) to study the specific composition of the entomological evidence in each case where *C. megacephala* was present; and (iii) to analyse the abiotic conditions and particularities of each case where it was recorded in order to draw conclusions about the role of this species as a forensic bioindicator. The results and conclusions aim to provide valuable new information for forensic investigations in the Iberian Peninsula and for future records in other European countries.

## Materials and methods

**Fig. 1:**
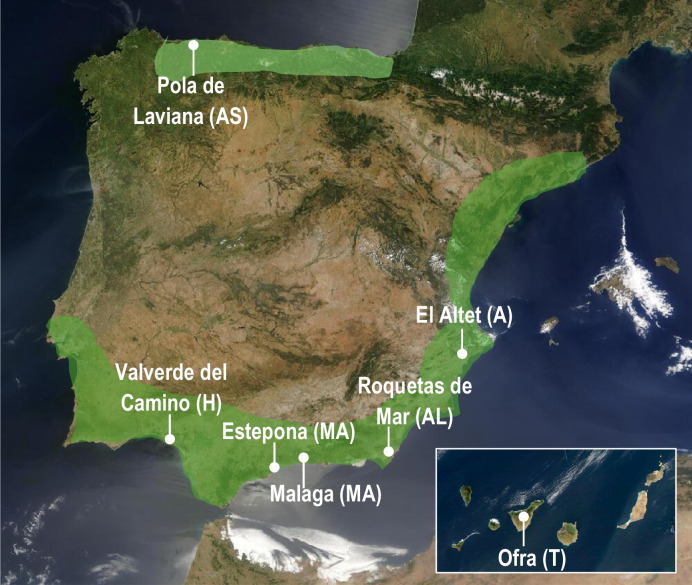
A map showing the potential distribution (shaded area) and new records on human corpses (white points) of *Chrysomya megacephala* in the Iberian Peninsula and Canary Islands. Abbreviations for provinces: A, Alicante; AL, Almería; AS, Asturias; H, Huelva; MA, Málaga; T, Tenerife

Entomological samples obtained during autopsies conducted at Institutes of Legal Medicine across various regions of Spain, killed and preserved directly in 70% ethanol, were sent to the reference centre INTCF (Madrid) for taxonomic identification by two authors of this manuscript (LMB, PMC and AMS). A review of all cases to date in which specimens of *C. megacephala* were collected was undertaken (Fig. 1). The specimens were deposited in the collection of the Laboratory of Biology at INTCF. Information for each case was provided by the forensic pathologist who performed the autopsy (Table 1). The average temperatures in each case were recorded from the time the individual was last seen alive until the autopsy, using data obtained from the meteorological station closest to the discovery location as provided by the State Meteorological Agency (AEMET, Spain).

**Table 1:** Summary of cases from ILMA and INTCF where *Chrysomya megacephala* was found breeding on human corpses. Includes case number, locality, biogeographic region, ambient where the corpse was found, type of death, dates related to the case, the average temperature from the time the individual was last seen alive to the autopsy, and the stage of decomposition as recorded by the forensic pathologist

Case number	Locality; coordinates; province; climate^1^	Biogeographic region^1^	Place and Habitat	Death	Disappear date	Autopsy date	Average Tª	Stage of decomposition
1·IMLA	El Altet; 38.2738, − 0.5398 DD; Alicante (SE Spain); Subtropical continental	Mediterranean	Indoor;	Accidental	Unknown	26/X/2022	22.2ºC	Bloated
2·INTCF	Roquetas de Mar; 36.7649, − 2.6155 DD; Almeria (SE Spain); Subtropical continental	Mediterranean	domestic Outdoor; camping area	Natural	26/VII/2020	1/VIII/2020	30.0ºC	Bloated
3·INTCF	Pola de Laviana; 43.2453, − 5.5611 DD; Asturias (N Spain); Subtropical maritime	Atlantic	Indoor; domestic	Natural	23/VI/2020	13/VII/2020	18.9ºC	Active decay
4·INTCF	Ofra, Tenerife; 28.4504, − 16.2926 DD; Canary Islands (Spain); Subtropical maritime	Macaronesian	Outdoor; mountain area	Violent	15/VIII/2022	5/IX/2022	30.0ºC	Active decay
5·INTCF	Valverde del Camino; 37.5747, − 6.7552 DD; Huelva (SW Spain); Subtropical maritime	Mediterranean	Indoor; domestic	Natural	2/VII/2020	1/VIII/2020	28.7ºC	Active decay
6·INTCF	Malaga city; 36.7217, − 4.4149 DD; Malaga (S Spain); Subtropical maritime	Mediterranean	Indoor; domestic	Natural	24/VIII/2020	30/VIII/2020	26.8ºC	Active decay
7·INTCF	Estepona; 36.4255, − 5.1513 DD; Malaga (S Spain); Subtropical maritime	Mediterranean	Indoor; domestic	Unknown	1/VII/2022	9/VII/2022	24.5ºC	Active decay

^1^ Climate and biogeographical region from https://www.eea.europa.eu/publications/report_2002_0524_154909

Larvae of *C. megacephala* and other mature Diptera larvae collected in all forensic cases were identified using the identification keys established by Velásquez et al. [16]. and Szpila [15], with binocular microscopes (Leica M80 and Leica MZ9.5). Previous studies were referenced for estimation of the age of the specimens based on the developmental periods and larval length [34, 36–42].

### Details of autopsy from forensic case 1·ILMA

**Fig. 2:**
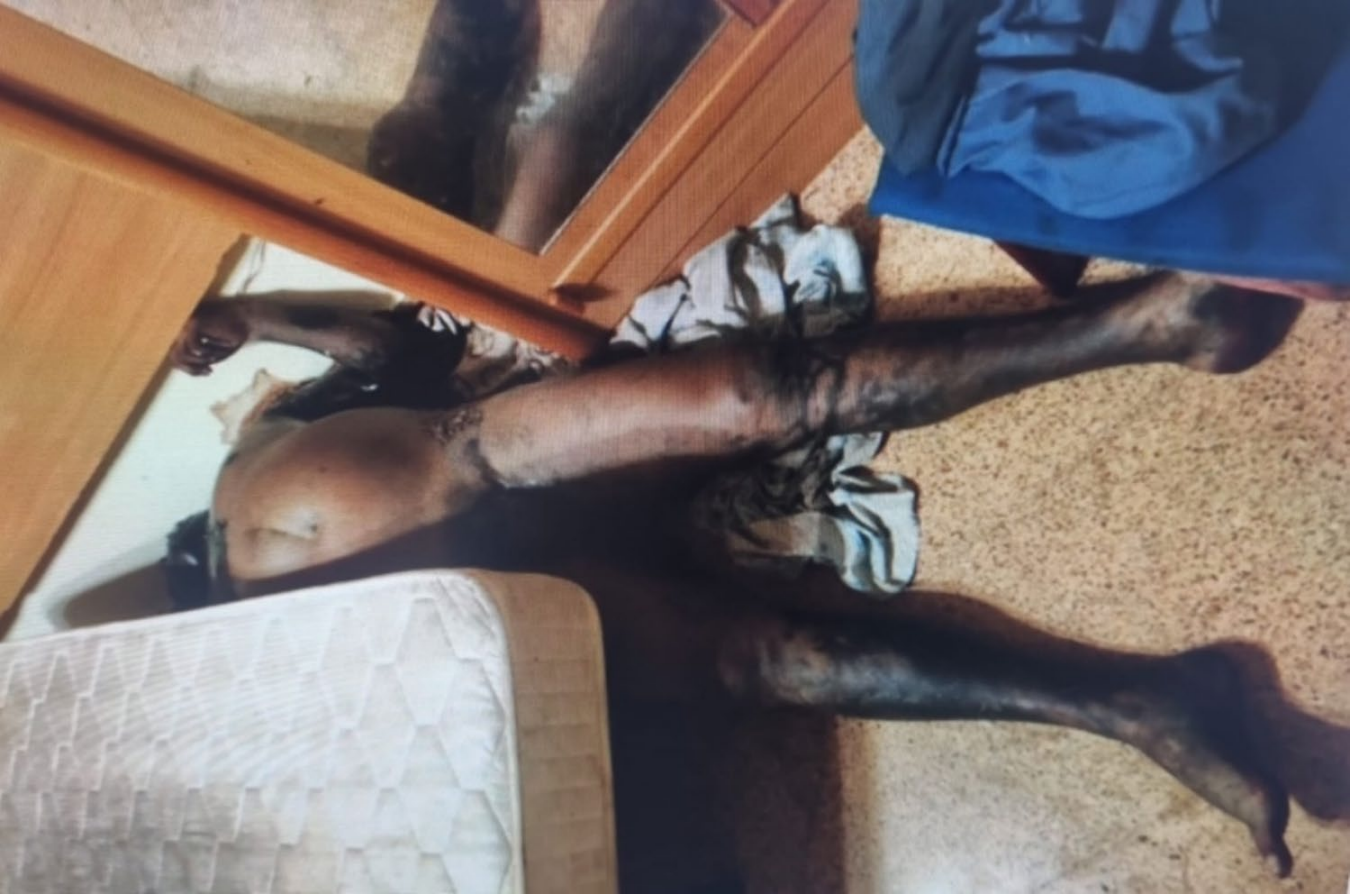
Case 1·ILMA, where the body of a man was found on the floor of his flat in El Altet (Alicante, southeast Spain)

After reviewing all the cases studied at the ILMA (Spain) from the last 20 years, specimens of *C. megacephala* were collected in only one autopsy. The body of a 62-year-old man was discovered on 25 October 2022 at 10:00 h inside his home in El Altet, a small coastal locality near Elche city, located in the province of Alicante (southeast Spain) (Fig. 1). The corpse was naked and found on the floor in a supine position, between the bed and the wall of the room. At the scene, remnants of tobacco and empty alcohol cans were found. The body was in an advanced stage of decomposition, exhibiting epidermal loss (Fig. 2). Police officers discovered the body after being notified by neighbours who had detected a strong odour. The door of the room remained closed, although windows were left open, likely because of the high temperatures in the preceding days.

The body was transferred to the ILMA facilities, where it was stored in a cold chamber at 5ºC for approximately 24 h. The autopsy was performed the following day (26 October 2022) at 10:00 h. The forensic pathologist determined that there was no evidence of violence at the time of death, and the death was estimated to have occurred 10 days earlier.

During the autopsy, specimens of the order Diptera were collected from the corpse by two authors of this manuscript (AMS and LCR) and immediately transferred to the laboratories at the University of Alicante (Alicante, Spain). The collected dipteran larvae were separated into two groups based on morphotypes and sizes. Half of the specimens were placed in boiling water for approximately 2 min, preserved in 70% ethanol for identification, and measured using an ocular micrometre adapted to a Leica M- 80 stereoscopic microscope (precision of 0.1 mm). The other half, including larvae and eggs, were reared at 24ºC ± 1.2ºC, 50% to 60% relative humidity, and a 12:12 light:dark cycle in a growth chamber in the laboratory, with liver provided ad libitum, following the methodology described by Ivorra et al. [35]. The samples were studied according to the recommended protocol for forensic entomology evidence collection [33].

The dates of pupation and adult emergence for each specimen were documented, along with the species, location on the corpse, and number of individuals, to establish the life cycle. The specimens were deposited in the Entomological Collection of the University of Alicante (Department of Environmental Sciences and Natural Resources). Meteorological data from the 10 days preceding the discovery of the corpse were recorded from the weather station closest to El Altet (Meteorological Station Altet-Elche Airport; data provided by AEMET, Spain). The mean daily maximum temperature was 27.2ºC ± 1.5ºC, the mean daily minimum temperature was 17.2ºC ± 1.6ºC, and the mean daily temperature was 22.2ºC ± 0.4ºC (Table 2).

**Table 2:** Meteorological data provided by AEMET from the Altet-Elche Airport weather station for the 10 days prior to the discovery of the corpse (*date of autopsy) in Case 1·ILMA, with the ADDs for *Chrysomya albiceps*

**Date**	**Day**	**Temperature (**^**o**^**C)**			**Humidity (%)**			**ADDs**
		**Max**	**Min**	**Average**	**Max**	**Min**	**Average**	**To *C. albiceps***
25/X/2022*	0	29.5	14.8	22.2	100	32	66	7.6
24/X/2022	1	26.6	17.5	22.0	100	53	76.5	15.1
23/X/2022	2	28.3	16.1	22.2	98	49	73.5	22.7
22/X/2022	3	24.8	19.2	22.0	100	76	88	30.2
21/X/2022	4	25.3	19.4	22.4	97	73	85	38.0
20/X/2022	5	26.8	18.7	22.8	96	67	81.5	46.3
19/X/2022	6	25.8	17.2	21.5	98	69	83.5	53.2
18/X/2022	7	27.2	17.2	22.2	97	60	78.5	60.9
17/X/2022	8	27.3	18.1	22.7	96	41	68.5	69.0
16/X/2022	9	29.0	15.4	22.2	92	36	64	76.7
15/X/2022	10	28.5	15.3	21.9	86	32	59	84.0
**Average** **SD**		27.2 1.5	17.2 1.6	22.2 0.4	96.4 4.1	53.4 16.6	74.9 9.5	

## Results

### Analysis of forensic cases

Six records of *C. megacephala* in mainland Spain and one from the Canary Islands are presented, based on a review of forensic cases from INTCF and ILMA (Table 1). The new data indicate the presence of *C. megacephala* in the Iberian Peninsula, in the north (Asturias), southeast (Alicante and Almería), south (Málaga), and southwest (Huelva) of Spain. All locations were near the coast, predominantly in southern regions (Fig. 1). The climate in these areas is subtropical, with the localities belonging to the Subtropical Maritime zone of the Mediterranean biogeographic region, where *C. megacephala* was most frequently observed. Regarding annual activity, *C. megacephala* was collected during the summer months, from late June to August, except for the case in Alicante (case 1·ILMA), where larvae were collected in the autumn, in October (Table 1). During these months, climatological conditions were mild and warm, with mean temperatures ranging from 20ºC to 30ºC. The exception was in Asturias (case 3·INTCF), where the mean temperature was 18.9ºC due to the climate in Atlantic biogeographic region (Table 1). High temperatures facilitate body decomposition, with five of seven cases reaching the active decay stage, according to information provided by forensic pathologists who performed the autopsies. In only two cases in southeastern Spain (Alicante, case 1·ILMA and Almería, case 2·INTCF) was the decomposition stage described as bloated. In both cases, larvae of *C. megacephala* in all instars were collected (Table 3). Finally, nearly all the bodies were found indoors in domestic settings, with deaths attributed to natural or accidental causes related to drug and alcohol abuse (Table 1). In the two outdoor cases, located in Almería (case 2·INTCF) and the Canary Islands (case 4·INTCF), the corpses were discovered in summer, in natural habitats under the highest mean temperature recorded in this study, 30ºC (Table 1).

**Table 3:** Forensic cases studied with the entomological evidence collected, showing the case number, the species collected, the stage and larval instars, maximum length of the largest larvae for each species, the immature age based on the constant temperature indicated. The postmortem interval estimated by forensic pathologist (PMI), the maximum PMI (maxPMI) based on the last time the individual was seen alive, and the minimum PMI (minPMI) based on entomological evidence and the oldest specimens (*)

Case	Species collected	Stage	Max. length	Immature age at temperature^1^	PMI/ maxPMI	minPMI
1·IMLA	*Chrysomya megacephala*	L2,3	12.4 mm	5 days; 22ºC	10 days / unknown	
	Chrysomya albiceps*	L2,3	17.7 mm	7.8 days; 21ºC		7.8 days
	*Calliphora vicina*	L3	18.5 mm	5.5 days; 22ºC		
	*Lucilia sericata*	L3	15.6 mm	4 days; 22ºC		
	*Synthesiomyia nudiseta*	L3	18.4 mm	6 days; 22ºC		
	Sarcophagidae	L3	18.4 mm	-		
2·INTCF	Chrysomya megacephala*	L1,2,3	18 mm	3 days; 30ºC	2.42 days / 6 days	3 days
	*Chrysomya albiceps*	L2,3	13 mm	2.3 days; 29ºC		
	*Lucilia sericata*	L2,3	15 mm	2,1 days; 30ºC		
	*Megaselia* sp	L?	4.5 mm	-		
	Sarcophagidae	L3	13 mm	-		
3·INTCF	*Chrysomya megacephala*	L3	14 mm	7.5 days; 19ºC	12 days / 19 days	
	Chrysomya albiceps*	L3	16 mm	14 days; 19ºC		14 days
	*Calliphora vicina*	L3	19.5 mm	7 days; 19ºC		
	*Lucilia sericata*	L2,3	15 mm	6 days; 19ºC		
4·INTCF	Chrysomya megacephala*	L3	9.5 mm	1.5 days; 30ºC	Unknown / 21 days	1.5 days
	*Lucilia sericata*	L3	9 mm	1.2 days; 30ºC		
5·INTCF	*Chrysomya megacephala*	L3	7 mm	1.5 days; 29ºC	30 days / 30 days	
	Chrysomya albiceps*	L1,2,3	10 mm	2 days; 29ºC		2 days
	*Lucilia sericata*	L3	11.5 mm	1.6 days; 29ºC		
	Sarcophagidae	L3	15 mm	-		
6·INTCF	*Chrysomya megacephala*	L3	13 mm	2.8 days; 27ºC	3–4 days / 6 days	
	*Chrysomya albiceps*	L2,3	10 mm	2.8 days; 27ºC		
	Calliphora vicina*	L3	17 mm	3 days; 27ºC		3 days
	Lucilia sericata *	L2,3	17.5 mm	3 days; 27ºC		
	Synthesiomyia nudiseta*	L3	15 mm	3 days; 25ºC		
7·INTCF	*Chrysomya megacephala*	L3	6.5 mm	1.7 days; 25ºC	7–8 days / 8 days	5.15–17.4^2^ days
	Lucilia sericata*	L3	18.5 mm	3.2 days; 24ºC		
	*Synthesiomyia nudiseta*	L3	9 mm	2.5 days; 25ºC		
	*Megaselia scalaris*	Pupa	-	> 5.15 days; 25ºC		

^1^Estimation of development days for each sample based on the literature referenced in the Materials and Methods and Results sections. ^2^Estimation of development days from egg to pupation and adult emergence

Regarding the entomological evidence, *C. megacephala* was recorded in the third instar larval stage in all seven cases and, also, in the first and/or second instar stages on the two corpses from southeastern Spain (cases 1·ILMA and 2·INTCF) (Table 3). No puparia were collected during the autopsies, possibly because mature larvae had dispersed before the bodies were recovered and because the entomological evidence was not sampled at the death scene. Other species belonging to the Calliphoridae, Muscidae, and Sarcophagidae families were sampled alongside *C. megacephala* during the autopsies (Table 3). In all cases, *C. megacephala* coexisted with mature larvae of *Lucilia sericata* (Meigen, 1826). *Chrysomya albiceps*, the only predator blow fly in Europe, was collected in all cases except for the Canary Islands, where the corpse was found in a natural habitat, and in one southern case at the beginning of summer (case 7·INTCF), where another predator, *Synthesiomyia nudiseta* (van der Wulp, 1883) (Diptera, Muscidae), was collected. The blue bottle blow fly *Calliphora vicina* Robineau-Desvoidy, 1830 was sampled in three of the seven cases: at the end of spring in the north (Asturias, case 3·INTCF), at the end of summer in the south (Málaga, case 6·INTCF), and at the beginning of autumn in the southeast (Alicante, case 1·ILMA), where the mean temperatures were 18.9ºC, 26.8ºC, and 22.2ºC, respectively (Table 1). Regarding the family Muscidae, *S. nudiseta* was the only species collected, coexisting with *C. megacephala* indoors in southeastern and southern Spain, i.e., Alicante and Málaga (cases 1·ILMA, 6·INTCF, and 7·INTCF), where the mean temperatures were 22.2ºC and 26.8ºC (Table 1). At the end of summer and early autumn, this muscid was sampled in cases with high species diversity, providing more potential prey. Other Diptera identified included larvae of Sarcophagidae, found in cases 2·INTCF and 5·INTCF (Table 3) during summer, when the highest mean temperatures were recorded in peninsular cases, and in case 1·ILMA from Alicante during autumn (Table 1). Finally, phorid scuttle flies were collected in July in nearby locations (Almería and Málaga) in southeastern and southern Iberian Peninsula, under outdoor and indoor conditions, respectively (cases 2·INTCF and 7·INTCF). In the latter case, a pupa of *Megaselia scalaris* (Loew, 1866) (Diptera, Phoridae) was identified.

In the cases where *C. megacephala* was recorded, the PMI estimated by the forensic pathologist ranged from 2 to 12 days except in case 5·INTCF, for which the PMI was impossible to determine. In this case, the PMI was estimated at 30 days before the autopsy based on the period since the individual was last seen alive, referred to as the maximum PMI (maxPMI). The maxPMI across all cases ranged from 6 to 30 days. Based on the entomological evidence and the oldest larvae collected, the minPMI ranged from 2 to 14 days, excluding the pupa of *M. scalaris* collected in case 7·INTCF (Table 3). Hence, *C. megacephala* could be considered a component of the first wave of colonisation. The age of the specimens collected was determined using measurements and larval growth curves at constant temperatures, as published previously for *C. megacephala* [34, 41], *C. albiceps* [42], *Ca. vicina* [40, 43], *L. sericata* [34, 36], *S. nudiseta* [39], and for *M. scalaris* [44]. In all cases, the size of *C. megacephala* larvae in the third instar ranged from 6.5 to 18.0 mm, and based on the growth curves at constant temperatures, their age ranged from 1.5 to 7.5 days. In two cases, 2·INTCF and 4·INTCF, *C. megacephala* was used to estimate the minPMI (3 days and 1.5 days, respectively) because the other blow flies, *C. albiceps* and *L. sericata*, arrived later as estimated by their age (Table 3). In case 6·INTCF, *C. megacephala* arrived almost simultaneously with other blow flies. In the remaining cases, the more developed specimens belonged to *C. albiceps*, *L. sericata*, and/or *Ca. vicina*, indicating that these species colonised the cadavers before *C. megacephala*. In cases where doubts arose, under the same temperature conditions, *C. megacephala* required less time than *L. sericata* and *C. albiceps* to reach a comparable stage of development, suggesting that the latter species could have been feeding on the corpses before *C. megacephala* (i.e., cases 5·INTCF). In general, *C. megacephala* could arrived earlier, simultaneously, or later than other necrophagous species.

### Estimation of minPMI from forensic case 1·ILMA

The case presented corresponds to 1·ILMA (Tables 1–3) and was autopsied at the ILMA in Alicante, Spain. Insect evidence was collected, preserved, reared, and identified as described in the methodology section. The sarcosaprophagous fauna found on the corpse consisted of third instar larvae from the Calliphoridae species *C. megacephala*, *C. albiceps*, *L. sericata*, and *Ca. vicina*, as well as Muscidae larvae of *S. nudiseta* and an unidentified larva of Sarcophagidae (the emerged female adult could not be identified) (Table 4). The most abundant species was *C. albiceps*, forming a large larval mass on the head, followed by *L. sericata*, *Ca. vicina*, *C. megacephala* and *S. nudiseta*, all of them considerably less abundant and located laterally on the thorax, avoiding the area where *C. albiceps* larvae were feeding. In terms of larval size, the longest larvae belonged to the blue bottle blow fly *Ca. vicina* and *S. nudiseta*, measuring 18.50 mm and 18.40 mm, respectively, but were present in low numbers (Table 4). Based on the mean temperature recorded at the nearest meteorological station, 22.2ºC ± 0.4ºC, *C. albiceps* required approximately 8 days to reach a length of 17.70 mm at 21ºC [42], *S. nudiseta* (18.40 mm) and *Ca. vicina* (18.50 mm) required approximately 6 days at 22ºC [39, 40, 43], and *C. megacephala* (12.40 mm) and *L. sericata* (15.60 mm) required 5 and 4 days at 22ºC, respectively [34, 36, 41] (Table 3). In conclusion, when the size of the larvae was used to estimate the minPMI, the age of the largest *C. albiceps* larvae indicated a time since first colonisation of approximately 8 days.

**Table 4:** Entomological evidence collected during the autopsy of Case 1·ILMA and preserved in 70% ethanol. Includes species, location on the corpse, larval instar, number of specimens preserved, and maximum length of the larvae collected

	**Specimens preserved**			
**Species**	**Location**	**Instar**	**No**	**Length**
*Chrysomya megacephala*	Lateral thorax	L3	10	12.4 mm
*Chrysomya megacephala*	Lateral thorax	L2	4	9.1 mm
*Chrysomya albiceps*	Head*	L3	36	17.7 mm
*Chrysomya albiceps*	Genitals	L2	16	5 mm
*Calliphora vicina*	Lateral thorax	L3	11	18.5 mm
*Lucilia sericata*	Lateral thorax	L3	19	15.6 mm
*Synthesiomyia nudiseta*	Lateral thorax	L3	2	18.4 mm
Sarcophagidae (no ident.)	Lateral thorax	L3	1	18.4 mm
Calliphoridae (no ident.)	Genitals	L2	76	4 mm

^*^ larval mass

Regarding the development of the evidential larvae at the laboratory of the University of Alicante under controlled conditions (24ºC ± 1.2ºC), the larvae of *C. albiceps*, *Ca. vicina*, and *S. nudiseta* were the first to pupariate, 2 days after the autopsy (Table 5). Based on the average temperature at which the corpse was found (22.2ºC), the time needed to reach pupariation at a constant temperature of 22ºC, according to previous literature (see methodology), is 10.4, 9.6, and 9.0 days, respectively (Table 5). Thus, colonisation occurred 8.4 days (10.4 days minus 2 days in the laboratory) before the corpse was discovered for *C. albiceps*, 7.6 days (9.6 days minus 2 days in the laboratory) for *Ca. vicina*, and 7 days (9 days minus 2 days in the laboratory) for *S. nudiseta*. Using the same method, *L. sericata* arrived 6 days before and *C. megacephala* at least 4.3 days before (Table 5).

**Table 5:** Species, number of specimens, minimal time of development (days) in the laboratory of the University of Alicante to the pupal stage (24ºC ± 1.2ºC) and from egg to pupa (at 22ºC) based on the literature, and estimation of the minPMI, for entomological evidence collected during the autopsy of Case 1·ILMA

**Species**	**No.**	**At laboratory: Time from larva collected to pupa (days)**	**Literature: Time from egg to pupa (days)**	**Estimation minPMI (days)**
*Chrysomya megacephala*	5	4	8.3	4.3
*Chrysomya albiceps*	40	2	10.4*	8.4
*Lucilia sericata*	53	3	9	6
*Calliphora vicina*	6	2	9.6	7.6
*Synthesiomyia nudiseta*	74	2	9	7

^*^for this species at 21ºC due to lack of data at 22ºC

Therefore, applying the life-cycle method, a minPMI of approximately 8 days was estimated based on the *C. albiceps* data, with similar results from preserved larvae (based on their length) and from reared larvae (based on the development period to pupariation). Finally, the accumulated degree-days (ADD) method was used to estimate the larval age of the pioneer species, *C. albiceps* (Table 2). With a minimum development threshold of 14.55ºC, calculated as the average threshold for egg and larval development until pupariation [42], *C. albiceps* requires 66.69 ADD at 22ºC. Based on the temperatures recorded in the days prior to the autopsy at the nearest meteorological station (Table 2), 60.9 ADD and 69.5 ADD were obtained for 7 and 8 days before the autopsy, respectively. Thus, 66.69 ADD corresponds to a period between 7 and 8 days prior to the autopsy. This result confirmed the previous minPMI estimation of 8 days based on larval measurement and development.

## Discussion

For the first time, *C. megacephala* has been reported on human corpses in Europe, specifically in six cases studied on the Iberian Peninsula and one in the Canary Islands. In all localities where this species was collected during autopsies, *C. megacephala* appeared to be confined to synanthropic locations mainly, near the coast across all latitudes, but primarily in the south and along the Mediterranean coast, consistent with previous reports of adults from Spain, Portugal, and North Africa [2, 3, 27, 45]. The spread of this species in Europe has largely followed the Mediterranean and Atlantic coastlines of the Iberian Peninsula, with specimens in this study collected during autopsies from Barcelona and Asturias, where adults had previously been observed on carrion and flowers [13, 14]. As with other exotic and synanthropic Diptera species with cosmopolitan or pantropical distributions, such as *Hermetia illucens* (Linnaeus, 1758) (Diptera: Stratiomyidae) and *S. nudiseta*, the Mediterranean region of Europe appears to be the most favourable for initial establishment on the continent [46, 47]. These non-native flies were first recorded in island territories such as Malta, the Canary Islands (Spain), and the Azores and Madeira Islands (Portugal). Approximately 20 to 30 years later, *S. nudiseta* and *H. illucens* were reported for the first time on mainland Europe, notably in southern Spain in locations such as Marbella, Valencia, and Alicante (at the Mediterranean basin; southern, southeastern, and eastern Spain). Since then, these species have gradually spread across western and central southern Europe, predominantly in Mediterranean countries [47, 48], while *H. illucens* has also expanded toward the Near East [49]. A similar process may have occurred with the spread of *C. megacephala*, which was first recorded in the Canary Islands in 1978 and on mainland Europe in 1998, specifically in Alicante (southeast Spain) [2, 11]. In Spain, the distribution and abundance of *C. megacephala* have increased over the past 25 years. The results of this study show records mainly from the east and south of the Iberian Peninsula but also in the north, where the Atlantic biogeographic region typically dominates. However, proximity to the coast likely facilitated its presence due to the mild meteorological conditions influenced by the sea. It is plausible that the distribution of *C. megacephala* will continue to expand in the Iberian Peninsula and, more broadly, in southern Europe, particularly in Mediterranean countries such as Portugal, France, Italy, and Greece. The apparent spread of this species along coastlines and islands suggests that maritime transport and commercial exchanges may have contributed to its introduction [11, 50], which may have occurred multiple times. For this reason, future records from other parts of Europe would not be surprising [45].

The larval activity of *C. megacephala* on human corpses was reported from June to October in indoor domestic habitats and, in two cases, outdoors in natural locations. The average temperatures during this period were warm, around 20ºC, but in the two outdoor cases, the average temperature was 30ºC. In the southeastern Iberian Peninsula, annual adult activity has been studied, beginning in spring (June) and continuing until autumn (November), with some specimens recorded in December in a peri-urban area between two well-populated localities (outdoors on the campus of the University of Alicante) [34]. However, the first European record was in the natural reserve of Clot de Galvany (Alicante, Spain), near well-populated localities [2]. In these reports, adults of *C. megacephala* were collected during the first 3 days of bait decomposition, mainly females attracted to lay eggs on the carrion [51]. In the autopsies analysed in this study, larvae of *C. megacephala* in all instars were collected from bloated corpses (cases 1·ILMA and 2·INTCF), while third instar larvae were found in corpses at the active decay stage, suggesting that this species might be considered a pioneer coloniser of human corpses in Spain. In experiments with animal models, where larvae were sampled daily, larvae of *C. megacephala* were collected for the first time the day 10 in autumn (12,94 ± 6,97ºC; HR media: 68,53 ± 24,49%), and the day 3 in summer (26,98 ± 6,88ºC; HR media: 63,66 ± 19,53%), and colonizing areas with lower larval concentration, avoiding competition with species such as *C. albiceps* and *L. sericata* [52]. This pattern was also observed in case 1·ILMA, where *C. megacephala* avoided the larval mass of *C. albiceps*. The timing of arrival of *C. megacephala* at carrion could be influenced by biotic and abiotic factors such as the condition of the corpse, the time of year, and the species composition of the necrophagous Diptera community. During the early years of its introduction, *C. megacephala* might be less abundant in the environment, and therefore, less likely than endemic species to encounter the odours of any specific corpse. High temperatures accelerate decomposition and lead to earlier fly arrival, as observed in cases 1·ILMA and 2·INTCF, where the average temperature was significantly high (30ºC). Supporting this observation in the timing of arrival of females to lay egg on the carrion, a recent experiment conducted in southeastern Spain during autumn, when temperatures were lower than in summer, found that *C. megacephala* did not appear until the seventh day of liver decomposition used as bait in traps; and in laboratory experiments, females laid more eggs on liver decomposed for 5—10 days than on liver fresh (*unpublished data*). It is well known that gravid female flies can retain eggs for extended periods in the absence of a suitable oviposition medium or under unfavourable conditions [53]. Furthermore, *C. megacephala* exhibits variable ovarian development rates depending on larval feeding, which can affect the timing of the first female cohort colonising carrion [54, 55]. Another factor to consider is maggot distribution and species-specific aggregation on carrion [56, 57], as the dispersal of larval masses limits the position of other necrophagous Diptera on cadavers; this was observed in case 1·ILMA. Therefore, more research is needed to investigate the colonisation behaviour of this species and its competitive interactions with other native species. Meanwhile, it can be concluded that this species, in real forensic cases, has the potential to act as a pioneer coloniser, consistent with observations in other parts of the world where *C. megacephala* arrives earlier than other *Chrysomya* species [21].

The species composition studied in the cases reported in this article is similar to that observed during spring, summer, and autumn in field studies conducted in the Iberian Peninsula [1], where the oriental latrine fly was collected alongside other primary colonising Diptera species, such as blow flies, flesh flies, and muscids. In Spain, *L. sericata* is the most common species in spring, *C. albiceps* dominates in summer, and in autumn; these species, together with *Ca. vicina*, are frequent and, coexist with *C. megacephala* in coastal provinces [52]. In the autopsies of our cases, *C. megacephala* was primarily collected from late spring to early autumn, avoiding the highest temperatures in each location, as was observed in Canary Island [11]. For instance, in the south of Spain at Mediterranean climate, temperatures can easily exceed 35ºC from mid-July to mid-August, whereas in June and September, they typically remain below of 25ºC. In the northern Atlantic region, temperatures in July and August generally stay below 30ºC, making *C. megacephala* more frequent during these months. Under such conditions, *C. megacephala* thrives in what resembles a tropical environment, with warm temperatures and relatively high humidity, often resulting from the characteristic spring and autumn rains of the Mediterranean climate. Based on its distribution in coastal provinces in the Mediterranean basin, *C. megacephala* could be a potentially useful indicator for postmortem relocation of cadavers.

Regarding the forensic cases reviewed, larvae of *C. megacephala* were captured alongside typical thermophilous species such as *C. albiceps* and *L. sericata*. In general, the corpses were in early decomposition stages, and the presence of these blow fly larvae in feeding and post-feeding phases indicated that flies had colonised the bodies recently. Notably, in several cases, larvae of *C. megacephala* in earlier instars were collected, but no puparia were found, most likely because of the absence of sampling around the body at the sites where the corpses were discovered. In all autopsies studied, *L. sericata* was present and coexisted with *C. megacephala*, suggesting that intraguild competition between the two species could occur, given their similar ecological requirements. However, the presence of both species as L3 larvae in all corpses suggests that, despite potential competition, they are able to coexist to the extent that both can produce the next generation from a single corpse. Nonetheless, the faster life cycle of *C. megacephala* may provide it with a competitive advantage. Previous study has shown that the life cycle of *L. sericata* is slower than that of *C. megacephala*, but that competition tends to impact *C. megacephala* more than the native species [34]. As such, fast larval feeding and rapid pupariation may be the best strategies for *C. megacephala* to avoid competition. When *C. megacephala* coexists with predators such as *C. albiceps* and *S. nudiseta*, it may avoid predation through strategies such as larval aggregation or accelerated pupariation [56, 58, 59]. Some authors have indicated that *C. megacephala* has greater tolerance to temperature variations than *C. albiceps*, which allows it to adapt better to variable climatic conditions. On corpses located in urban habitats, particularly domestic settings, the coexistence of these three species is common, with *C. albiceps* or *L. sericata* often serving as forensic indicators of the minPMI. And, in outdoor conditions, such as cases 2·INTCF and 4·INTCF, *C. megacephala* is well adapted and serves as an indicator of the minPMI, demonstrating its pioneer role in corpse colonisation. This aligns with findings from other parts of the world, where *C. megacephala* is ubiquitous and a key species for estimating the minPMI [21]. In both cases, *C. megacephala* arrived before *L. sericata* and *C. albiceps*, with the minPMI estimates based on its larval age being 3 days and 1.5 days, respectively, reflecting its rapid development. In some cases, the predator muscid *S. nudiseta* was also collected with *C. megacephala*. Interestingly, this muscid appeared in cases with high species diversity, likely due to its predatory behaviour [58]. *Synthesiomyia nudiseta* is a pioneer species and appeared in all forensic cases during the early stages of decomposition, although it arrived slightly later than other necrophagous species such as *L. sericata* and *C. albiceps*, but earlier than *C. megacephala*, as estimated by maggot age analysis. Another necrophagous blow fly collected in the studied cases was *Ca. vicina*, which was recorded during the first month of autumn and the last months of spring, indicating its adaptation to cooler conditions typical of *Calliphora* species [1]. This species was found in cases with the highest diversity of forensic Diptera, coexisting with two other necrophagous species (*C. megacephala*, *L. sericata*) and two predators (*C. albiceps* and *S. nudiseta*). The specifics of interspecific competition in these contexts remain unknown. Intraguild competition on carrion has been studied previously, including interactions between *L. sericata* and *C. megacephala* in European populations [34, 35, 47, 58–60]. However, further studies are needed to include all species and to investigate the effects of intraguild competition under different conditions.

In the Case 1·ILMA the location of the *C. albiceps* larval mass on the head corresponds to the natural pattern of colonisation in orifices such as the eyes, nostrils, mouth, and ears. Simultaneously, the presence of third instar larvae of *L. sericata*, *Ca. vicina*, *S. nudiseta*, *C. megacephala*, and flesh flies on the lateral areas of the body and arm suggests that these species might have been avoiding predation by *C. albiceps*. These species were observed leaving the body, a stage known as the prepupal stage. Notably, *Chrysomya* species were the first to complete their life cycle in the laboratory, with the minPMI estimated at 8 days based on the development of *C. albiceps* until pupariation, 7.8 days based on larval length, and 7–8 days using the ADD method. *Chrysomya megacephala* arrived later than *C. albiceps*, *S. nudiseta*, *Ca. vicina*, and *L. sericata*, based on the time to pupariation; however, its larval length indicated that it was older than *L. sericata*. This discrepancy could be explained by the developmental differences at 22ºC: *C. megacephala* requires 8.3 days to pupariate, whereas *L. sericata* requires 9.0 days. In this case, if the minPMI was estimated at 7 to 8 days using *C. albiceps*, it is plausible that some specimens of *C. megacephala* had already left the corpse to pupariate. However, the death scene was not sampled by the forensic pathologist or scientific police, leaving it unknown whether puparia of *C. megacephala* were present in the room. These findings suggest that specimen collection may have been incomplete. In conclusion, it is recommended to collect specimens at the discovery scene [33] and apply all available methods to estimate the minPMI (e.g., length, life cycle, and ADD method) because pupae may have left the corpse to pupariate and could be present at the scene but not on the corpse.

The oriental latrine fly was discovered in Europe in 1998 [2], but it had not been recorded on human corpses in this continent until now. This study reports its presence since 2020 in the north, south, and southeast of Spain, indicating that the species is well established across the coastal areas of the Iberian Peninsula. It is possible that larvae of this species were previously overlooked or misidentified as other blow fly species, such as *Ca. vomitoria* (Linnaeus, 1758), because of their similar characteristics: large posterior spiracles, an oral sclerite at least partly sclerotised, and large, robust spines that are strongly sclerotised with single or serrated tips, arranged separately rather than in short rows [17]. Other necrophagous flies of forensic importance have also been overlooked in Europe in the past decade, such as *S. nudiseta*. This muscid may have been misidentified because its larvae resemble those of *Musca* *domestica *Linnaeus, 1758, and its adults are similar to *Muscina* Robineau-Desvoidy, 1830, or certain flesh fly species. Moreover, *S. nudiseta* had not been described in forensic cases in Europe before the study by Velásquez et al. [39], and until 2010, there were no published identification keys from European territory to identify it [16]. The case of *C. megacephala* is similar to that of *S. nudiseta*, although it is possible to identify adults using morphological keys from other regions of the world, even though the composition of the Diptera community in southwestern Europe may differ. In Europe, there are only a few taxonomic studies in which *C. megacephala* was included, such as the works on adults from the Canary Islands [10, 19], and the studies by Szpila [15] and Velásquez et al. [16]. on southwestern blow fly keys for larvae. However, it is not possible to identify larvae of Spanish *Chrysomya* species, including those from the Canary Islands (*C. albiceps*, *C. putoria*, and *C. megacephala*), using European identification keys; only keys from Africa or America, such as those by Szpila et al. [17], are applicable.

## Conclusions

*Chrysomya megacephala* is an important potential forensic indicator in Spain. This species arrives on corpses alongside other pioneer species, primarily *L. sericata* and *C. albiceps*, and its rapid development makes it suitable for estimating short values of the minPMI. It is likely a pioneer coloniser of human corpses and is expected to establish itself further in mainland Europe in the coming decades. Data on its biology and ecology, obtained through laboratory experiments and field studies, will be crucial for understanding its interactions with other necrophagous Diptera in Europe. Such information can be applied to real forensic cases in the future, aiding in the estimation of the time of colonisation on corpses and confirming their location, which is typically near the coast, mainly in urban indoor settings but also in outdoors or in semi-natural environments.

## Supplementary Information

Below is the link to the electronic supplementary material.

Supplementary file1 (DOCX 22 KB)

## Data Availability

Entomological evidence collected during the autopsy and reared in the laboratory, at the case 1·ILMA.
