# Correction to: The oriental latrine fly *Chrysomya* *megacephala* (Fabricius, 1794) (Diptera: Calliphoridae) as a new forensic indicator in SW Europe

**DOI:** 10.1007/s00414-025-03508-z

**Published:** 2025-05-03

**Authors:** Anabel Martínez-Sánchez, Tania Ivorra, Leticia C. Roberts, Salvador Giner, Luisa M. Beringola, Pedro M. Cano, Santos Rojo

**Affiliations:** 1 Department of Environmental Sciences and Natural Resources, University of Alicante, PO Box 99, 03080 Alicante, Spain; 2 Department of Parasitology, Faculty of Medicine, University Malaya, 50603 Kuala Lumpur, Malaysia; 3 Institute of Legal Medicine of Alicante, Pathology Service, Generalitat Valenciana, 03006 Alicante, Spain; 4 National Institute of Toxicology and Forensic Sciences, Ministerio de la Presidencia, Justicia y Relaciones con las Cortes, 28232 Las Rozas de Madrid, Madrid Spain

**Correction to: International Journal of Legal Medicine**

10.1007/s00414-025-03489-z

In the original version of the manuscript, there was a typographical error in co-author Tania Ivorra's name, mistakenly written as “Tania E. Ivorra”. It should not include a middle name.

The correct author name is Tania Ivorra, not Tania E. Ivorra.

The original article has been corrected.

