# Supplementary material for: The oriental latrine fly Chrysomya megacephala (Fabricius, 1794) (Diptera: Calliphoridae) as a new forensic indicator in SW Europe
Source: Int J Legal Med. 2025 Apr 12;139(5):2425–37. doi: 10.1007/s00414-025-03489-z (PMC12354500; doi:10.1007/s00414-025-03489-z)
Supplement: Supplementary file 1 — Supplementary file1 (DOCX 22 KB) [file 414_2025_3489_MOESM1_ESM.docx]

**Supplementary Data**

Entomological evidence collected during the autopsy and reared in the laboratory, at the case 1·ILMA. For each species, dates to the first specimens reached the pupal and to adult stages, and days from the date of specimen collection during the autopsy (*starting from 26 October 2022, inclusive).

| **Species**  **collected and reared** | **Localization on the corpse** | **Stage; instar** | **Date of pupation** | **Date of emergence** | **Days to pupa*** | **Days to adult*** | **No** |
| --- | --- | --- | --- | --- | --- | --- | --- |
| *Calliphora vicina* | Thorax | L3 | 28/X/22 | 06/XI/22 | 2 | 11 | 1 |
| *Calliphora vicina* | Arm | L3 | 29/X/22 | 06/XI/22 | 3 | 11 | 1 |
| *Calliphora vicina* | Thorax | L3 | 29/X/22 | 08/XI/22 | 3 | 13 | 2 |
| *Calliphora vicina* | Thorax | L3 | 28/X/22 | 09/XI/22 | 2 | 14 | 2 |
| *Chrysomya albiceps* | Head | L3 | 28/X/22 | 03/XI/22 | 2 | 8 | 17 |
| *Chrysomya albiceps* | Head | L3 | 29/X/22 | 03/XI/22 | 3 | 8 | 13 |
| *Chrysomya albiceps* | Thorax | L3 | 30/X/22 | 04/XI/22 | 4 | 9 | 1 |
| *Chrysomya albiceps* | Thorax | L3 | 30/X/22 | 04/XI/22 | 4 | 9 | 1 |
| *Chrysomya albiceps* | Arm | L3 | 30/X/22 | 05/XI/22 | 4 | 10 | 6 |
| *Chrysomya albiceps* | Hand | Eggs |  |  |  |  | >300 |
| *Chrysomya megacephala* | Thorax | L3 | 30/X/22 | 04/XI/22 | 4 | 9 | 2 |
| *Lucilia sericata* | Thorax | L3 | 29/X/22 | 06/XI/22 | 3 | 11 | 5 |
| *Lucilia sericata* | Genitals | ¿ | 29/X/22 | 06/XI/22 | 3 | 11 | 3 |
| *Lucilia sericata* | Thorax | L3 | 30/X/22 | 06/XI/22 | 4 | 11 | 36 |
| *Lucilia sericata* | Arm | L3 | 30/X/22 | 06/XI/22 | 4 | 11 | 7 |
| *Lucilia sericata* | Thorax | L3 | 30/X/22 | 08/XI/22 | 4 | 13 | 2 |
| *Sarcophaga* (female) | Thorax | L3 | 29/X/22 | 14/XI/22 | 3 | 19 | 1 |
| *Synthesiomyia nudiseta* | Thorax | L3 | 29/10/22 | 08/11/22 | 3 | 13 | 1 |
| *Synthesiomyia nudiseta* | Thorax | L3 | 29/X/22 | 09/XI/22 | 3 | 14 | 2 |
| *Synthesiomyia nudiseta* | Arm | L3 | 29/X/22 | 09/XI/22 | 3 | 14 | 1 |
| *Synthesiomyia nudiseta* | Thorax | L3 | 29/X/22 | 11/XI/22 | 3 | 16 | 1 |
| *Synthesiomyia nudiseta* | Thorax | L3 | 29/X/22 | 12/XI/22 | 3 | 17 | 1 |
